# Quantitative Comparison of PET and Bremsstrahlung SPECT for Imaging the In Vivo Yttrium-90 Microsphere Distribution after Liver Radioembolization

**DOI:** 10.1371/journal.pone.0055742

**Published:** 2013-02-06

**Authors:** Mattijs Elschot, Bart J. Vermolen, Marnix G. E. H. Lam, Bart de Keizer, Maurice A. A. J. van den Bosch, Hugo W. A. M. de Jong

**Affiliations:** Department of Radiology and Nuclear Medicine, University Medical Center Utrecht, Utrecht, The Netherlands; University of Modena & Reggio Emilia, Italy

## Abstract

**Background:**

After yttrium-90 (^90^Y) microsphere radioembolization (RE), evaluation of extrahepatic activity and liver dosimetry is typically performed on ^90^Y Bremsstrahlung SPECT images. Since these images demonstrate a low quantitative accuracy, ^90^Y PET has been suggested as an alternative. The aim of this study is to quantitatively compare SPECT and state-of-the-art PET on the ability to detect small accumulations of ^90^Y and on the accuracy of liver dosimetry.

**Methodology/Principal Findings:**

SPECT/CT and PET/CT phantom data were acquired using several acquisition and reconstruction protocols, including resolution recovery and Time-Of-Flight (TOF) PET. Image contrast and noise were compared using a torso-shaped phantom containing six hot spheres of various sizes. The ability to detect extra- and intrahepatic accumulations of activity was tested by quantitative evaluation of the visibility and unique detectability of the phantom hot spheres. Image-based dose estimates of the phantom were compared to the true dose. For clinical illustration, the SPECT and PET-based estimated liver dose distributions of five RE patients were compared. At equal noise level, PET showed higher contrast recovery coefficients than SPECT. The highest contrast recovery coefficients were obtained with TOF PET reconstruction including resolution recovery. All six spheres were consistently visible on SPECT and PET images, but PET was able to uniquely detect smaller spheres than SPECT. TOF PET-based estimates of the dose in the phantom spheres were more accurate than SPECT-based dose estimates, with underestimations ranging from 45% (10-mm sphere) to 11% (37-mm sphere) for PET, and 75% to 58% for SPECT, respectively. The differences between TOF PET and SPECT dose-estimates were supported by the patient data.

**Conclusions/Significance:**

In this study we quantitatively demonstrated that the image quality of state-of-the-art PET is superior over Bremsstrahlung SPECT for the assessment of the ^90^Y microsphere distribution after radioembolization.

## Introduction

Intra-arterial radioembolization (RE) using microspheres labeled with the high-energy beta-emitter yttrium-90 (^90^Y), is used in clinical practice for treatment of unresectable liver tumours [1], [2]. Prior to RE, prophylactic coil-embolization of arteries communicating with the gastrointestinal (GI) tract is performed, followed by administration of technetium-99m macroaggregated-albumin (^99m^Tc-MAA) particles. Subsequent gamma camera imaging is used to assess any extrahepatic particle deposition, which is a contra-indication for RE. Also, the percentage of particles that shunts to the lungs is measured, which may lead to adjustment of the ^90^Y dose [3]. After administration of the ^90^Y microspheres, a post-therapy ^90^Y Bremsstrahlung SPECT scan is performed for two reasons. First, ^90^Y may unexpectedly be present outside the liver, despite a favorable distribution on the ^99m^Tc-MAA scan. Accumulation of ^90^Y microspheres in organs other than the liver will likely cause serious complications, like ulceration and bleedings in the GI tract [4], [5]. Therefore, severe pain after treatment should be aggressively managed to prevent development of more serious complications [5], which may be facilitated by early detection of possible extrahepatic activity [6]. Second, the intrahepatic microsphere distribution over tumourous and non-tumourous liver tissue is expected to be an important predictor of treatment efficacy [7]. Post-therapy imaging facilitates estimation of the tumour and non-tumour absorbed radiation dose on the image-based microsphere distribution.

Unfortunately, the low photon yield and continuous nature of the Bremsstrahlung X-ray spectrum limit the quantitative accuracy of ^90^Y Bremsstrahlung SPECT. Per MBq, approximately 23000 Bremsstrahlung photons with energy higher than 50 keV are produced by interaction of the beta particle with tissue [8], therefore acquisition in a wide energy window is required to maximize sensitivity. The absence of a photopeak prohibits the use of simple window-based scatter rejection, scatter correction, and attenuation correction techniques, and penetration of high-energy photons through the collimator septa leads to further loss of image contrast [9]. Recent efforts to optimize reconstruction algorithms, e.g. by compensation for scatter and collimator blurring with pre-calculated ^90^Y point-spread functions (PSF) [8], [10], have improved the quantitative accuracy of ^90^Y Bremsstrahlung SPECT, but are not widely available in the clinic to date.

Yttrium-90 PET was recently shown to be feasible in phantoms and patients and may be an interesting alternative to Bremsstrahlung SPECT [11]. Per MBq, ^90^Y emits 32 positrons per second with a maximum energy of 758 keV [12], [13]. Consequently, annihilation photon pairs are produced approximately 700 times less often than Bremsstrahlung photons (E >50 keV). Nevertheless, ^90^Y PET may allow for the detection of extrahepatic activity and accurate tumour and liver dose estimation, since the spatial resolution is expected to be comparable to that of ^18^F PET, which has a maximum positron energy of 633 keV [14]. Additionally, advanced correction techniques for scatter, random, and attenuation effects that are clinically available for ^18^F PET, can be directly applied to ^90^Y PET [15]. Several groups have successfully assessed the post-therapy ^90^Y microsphere distribution in RE patients [11], [16]–[19].

To date, no systematic studies have quantitatively compared clinically available ^90^Y PET with Bremsstrahlung SPECT. The potential for accurately assessing the intra- and extrahepatic microsphere distribution is determined by fundamental limitations of the imaging modality. The first objective of this study was to quantitatively evaluate and compare the contrast and noise characteristics of ^90^Y PET and SPECT images, which are relevant parameters for activity detection and liver dosimetry in clinical practice. Image contrast depends on spatial resolution and on other effects that distort quantification of local ^90^Y content, like photon attenuation, photon scatter, collimator penetration and random coincidences. Image noise is dependent on sensitivity and on the stochastic nature of attenuation, scatter and collimation. The second objective was to quantitatively compare ^90^Y PET and SPECT on the ability to detect small accumulations of ^90^Y inside and outside the liver, and on the accuracy of liver dose estimates, based on the recorded ^90^Y distribution. All quantitative results were obtained in phantom experiments. Additionally, Bremsstrahlung SPECT and PET scans of five ^90^Y RE patients were evaluated to support the results of the phantom study.

## Materials and Methods

### Ethics Statement

Dual post-treatment imaging using both SPECT/CT and PET/CT techniques was part of the quality control protocol for implementation of ^90^Y-PET imaging as the standard post-radioembolization procedure. All data were retrospectively analyzed, written patient informed consent was therefore not sought nor documented. The institutional review board (IRB) of the University Medical Center Utrecht approves this type of retrospective study and waives the requirements for patient informed consent. The image data were handled anonymously, in accordance with the Declaration of Helsinki and the regulations of the IRB.

### Scanners, Acquisition and Reconstruction

PET/CT data were acquired on a state-of-the-art Siemens mCT Time-Of-Flight (TOF) PET/CT scanner (Siemens Medical Solutions USA, Inc.). The mCT combines a whole body LSO PET scanner with a 40-slice CT scanner. The PET component is equipped with a large axial field-of-view (FOV) of 21.8 cm and wide acceptance angle of 13.2° to maximize sensitivity. In whole body mode, consecutive bed positions overlap approximately 43%. A detailed characterization of the physical and clinical performance of the mCT was performed by Jakoby *et al* [20]. PET data were acquired with a lower- and upper-level discriminator of 435 keV and 650 keV, respectively. Three reconstruction algorithms were compared, all including correction for random coincidences, scatter and attenuation [20]. The first algorithm was fully 3D iterative attenuation weighted ordinary Poisson ordered subset expectation maximization (OP-OSEM) reconstruction [21] (‘iterative’), the second algorithm was iterative reconstruction extended with a PSF model of the detector response [22] (‘iterative+PSF’), and the third was iterative reconstruction including PSF and TOF information (‘iterative+PSF+TOF’). Images were reconstructed using 3 iterations with 24 or 21 (iterative+PSF+TOF) subsets and a 5 mm full-width at half-maximum (FWHM) Gaussian post-reconstruction filter. The reconstructed voxel size was 2×2×2 mm^3^.

SPECT/CT data were acquired on a Siemens Symbia T16 system (Siemens Medical Solutions USA, Inc.). The Symbia T16 combines a dual detector SPECT scanner with a 16-slice CT scanner. The SPECT detectors are equipped with 3/8 inch thick crystals. All our experiments were performed with the high-energy collimators mounted, which proved to be the best option for ^90^Y imaging with maximum resolution and contrast [9], . SPECT data were acquired in a 105–195 keV energy window [8], [24], using 120 projections over 360° and a 256×256 matrix with a pixel size of 2.4×2.4 mm^2^. Images were reconstructed with two different reconstruction algorithms. The first algorithm was 2D OSEM reconstruction including correction for attenuation with an effective broad beam linear attenuation coefficient (‘iterative’), and the second algorithm was iterative reconstruction including a Gaussian PSF model of the geometric collimator-detector response of the camera [25] (‘iterative+PSF’). In both cases 8 iterations with 8 subsets were used without post-reconstruction filtering. The reconstructed voxel size was 2.4×2.4×2.4 mm^3^.

### Phantom Set Up

The NEMA 2007/IEC 2008 PET Image Quality (IQ) Phantom, which is designed for evaluation of reconstructed image quality and activity recovery, was used for all phantom experiments to allow for direct comparison between the two modalities [26]. The phantom consists of a fillable torso-shaped compartment (volume = 9700 ml) containing six fillable coplanar spheres (inner diameter = 10, 13, 17, 22, 28, and 37 mm) and a cylindrical lung insert.

Table 1 lists the separately performed phantom experiments. PET data were acquired in one bed position centered on the spheres. Since the PET FOV covering a whole liver requires two bed positions (30 cm in axial direction) and for SPECT one FOV (40 cm) is sufficient, all PET scan times were set to half the SPECT scan times. The initial ^90^Y activity concentration in the spheres ([A]_sph_) and background compartment ([A]_bkg_) was 2.40 and 0 MBq ml^−1^, respectively. All six spheres were filled with activity, which slightly deviates from the NEMA recommendations. After performing experiments 1, ^90^Y was added to the background for experiments 2–4, resulting in [A]_bkg_ = 0.27 MBq ml^−1^. The total activity in the phantom was 119 and 2600 MBq, for experiments 1, and 2–4, respectively. Instead of adjusting the activity in the phantom, the scan times were prolonged ×2 and ×4 times in experiments 2–4 to obtain the simulated activity concentrations given in Table 1. This approach was deemed valid, because both the measured total activity and the maximum simulated total activity, which corresponded to approximately 2600 MBq for a 2000 ml liver, were within the linear range (i.e. no dead time effects) of ^90^Y PET and Bremsstrahlung SPECT [9], .

**Table 1:** Phantom experiments.

Exp.	Ratio	Modality	Real scan time (min.)	[A]_sph_ (MBq ml^−1^)	[A]_bkg_ (MBq ml^−1^)
1P	1∶0	PET	15	2.4 (real)	0 (real)
1S	1∶0	SPECT	30	2.4 (real)	0 (real)
2P	9∶1	PET	15	2.4 (real)	0.27 (real)
2S	9∶1	SPECT	30	2.4 (real)	0.27 (real)
3P	9∶1	PET	30	4.8 (simulated)	0.54 (simulated)
3S	9∶1	SPECT	60	4.8 (simulated)	0.54 (simulated)
4P	9∶1	PET	60	9.6 (simulated)	1.09 (simulated)
4S	9∶1	SPECT	120	9.6 (simulated)	1.09 (simulated)

### Image Contrast and Noise

Image contrast and noise measurements were obtained from phantom experiments 4 (Table 1), representing a clinically realistic liver background activity concentration of 1.09 MBq ml^−1^. For comparison: using the body surface area (BSA) method (see ‘Patients’ section), the mean activity concentration in a 1.5 kg liver with 10% tumour involvement of an ‘average’ patient (1.70 m, 70 kg) would be 1.14 MBq ml^−1^. Six circular hot sphere regions of interest (ROIs) were automatically delineated on the CT slice through the center of the spheres, covering the inner compartment voxels of each of the six phantom spheres. A background ROI was delineated on the same slice consisting of all voxels that fell within the physical phantom boundaries, excluding the voxels belonging to the hot sphere ROIs and the lung insert. For each reconstructed SPECT and PET volume, contrast recovery coefficients (Q_H_) were calculated with equation 1.
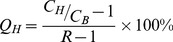
(1)Here, C_H_ is the mean intensity in the hot sphere ROIs, C_B_ is the mean intensity in the background ROI and R is the true sphere-to-background activity concentration ratio. As a measure of image noise, the coefficient of variation (CV) was calculated with equation 2.
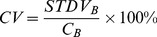
(2)Here, STDVB is the standard deviation in the background ROI.

### Detectability

The ability to detect small accumulations of activity (detectability) in a region without background activity is important for evaluation of extrahepatic microsphere deposition. Additionally, the detectability of activity in a region with background activity is important when intrahepatic tumour identification is based on the contrast in the SPECT and PET scans, e.g. using isocontours when the target volume cannot be derived from a CT or MRI scan.

A hot spot of activity can only be accurately identified, if it can be reliably distinguished from the background, i.e. if it is visible. The visibility (υ_H_) of the six hot spheres was tested by quantitative evaluation of the hot sphere and background signal; for each hot sphere the effective contrast-to-noise ratio (CNR) was calculated according to equation 3.
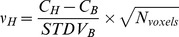
(3)with C_H_, C_B_, and STDV_B_ as defined in the previous section and N_voxels_ the number of voxels in the hot sphere ROI. Subsequently, a hot sphere was scored ‘visible’ when υ_H_ was larger than 4, in accordance with the Rose criterion [27], [28].

In addition to the visibility criterion, which is an established method for detection of false negatives, a new method is presented to evaluate the presence of false positive hot spots. Since accumulations of activity are not ‘uniquely detectable’ if false positives are present, we will refer to this criterion as ‘unique detectability’ in the remainder of this work. The unique detectability of the six hot spheres was evaluated in five consecutive background slices, placed five cm away from the slice through the center of the spheres. Circular ROIs to test for false positives (ROI_TEST-FP_) with the same diameter as the hot sphere ROIs were centered on all phantom voxels in the five slices. The visibility (υ_TEST-FP_) of each ROI_TEST-FP_ was calculated with equation 4.
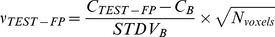
(4)Here, C_TEST-FP_ is the mean intensity in ROI_TEST-FP_, C_B_ the mean intensity in the background ROI of the same slice (consisting of all voxels falling within the phantom boundaries, excluding the lung insert), STDV_B_ the standard deviation in the background ROI and N_voxels_ the number of voxels in ROI_TEST-FP_. We investigated two different criteria to score a sphere ‘uniquely detectable’. According to the first criterion, a visible hot sphere was scored ‘uniquely detectable’, if υ_TEST-FP_ was smaller than 4 for all ROI_TEST-FP_ of the same size, i.e. if none of the corresponding ROI_TEST-FP_ were visible according to the Rose criterion. Translated to clinical practice, this criterion measures whether any visible hot spot of a certain diameter can be reliably characterized as ‘true positive’. According to the second criterion, a visible hot sphere was scored ‘uniquely detectable’ if υ_TEST-FP_ was smaller than υ_H_ for all ROI_TEST-FP_ of the same size, i.e. if the hot sphere was better visible than all corresponding ROI_TEST-FP_. This criterion is less strict and measures whether the visible hot spot with the highest CNR can be reliably characterized as ‘true positive’.

When using CNR with the relaxation term √N_voxels_ for evaluation of the visibility of hot spots, a uniform count density in the hot spot ROI is assumed. However, the mean intensities of the relatively small hot sphere and false positive ROIs are susceptible to outliers and may therefore not accurately represent human perception. Median values, on the other hand, are less sensitive to outliers and may be a better measure for this purpose. Consequently, equations 3 and 4 were also evaluated using median intensities for C_H_ and C_TEST-FP_, respectively. The ‘median-based’ hot sphere visibility and unique detectability results were presented next to their ‘mean-based’ equivalents. Data from experiments 1 (Table 1) were used to analyze the extrahepatic detectability and experiments 2–4 were used to mimic the intrahepatic situation, for three different activity concentrations.

### Patients

Radioembolization with ^90^Y-labelled resin microspheres (SIR-Spheres, Sirtex Medical, Sydney, Australia) was performed in 5 consecutive patients (2 women, 3 men; mean age +/− SD, 56.8+/−13.0 y; age range 43–76 y) according to the international consensus report from the Radioembolization Brachytherapy Oncology Consortium [3]. The BSA method was used to calculate the activities (A) administered to the patients, according to the equation A (GBq) = BSA−0.2+ V_T_/V_L_, with BSA (m^2^) = 0.20247 * height^0.725^ * weight^0.725^, and V_T_ and V_L_ the tumour and whole liver volume derived from CT or MRI, respectively. Dose adjustments were not required, since the percentage ^99m^Tc-MAA lung shunting was below 10% for all patients. Four patients were diagnosed with liver metastases (2 colorectal carcinomas, 1 nasopharyngeal carcinoma, and 1 mammary carcinoma) and one patient was diagnosed with hepatocellular carcinoma. Four patients received whole liver treatment and one patient received left lobar treatment. PET data were acquired in two bed positions (total scan length 30 cm) centered on the liver, whereas the SPECT axial FOV was 40 cm. SPECT and PET data were both acquired in 30 minutes, which was considered the maximum desirable scan time for a RE patient. All patient and treatment characteristics are summarized in Table 2.

**Table 2:** Patient and treatment characteristics.

Patient	Primary^a^	Lobes^b^	V_L_ c	V_T_ d	A_A_ e	A_S_ f	A_P_ g
1	CRC	W	2481	153	1714	1303	1602
2	CRC	W	1835	50	1852	1483	1499
3	NPC	W	1230	72	1199	922	1109
4	MC	L	383	20	281	216	270
5	HCC	W	3050	178	1177	916	927

a Primary disease: CRC = colorectal carcinoma; NPC = nasopharyngeal carcinoma; MC = mammary carcinoma; HCC = hepatocellular carcinoma.

b Lobes treated: W = whole liver treatment; L = left lobar treatment.

c V_L_ = treated liver volume (ml).

d V_T_ = volume of tumours in treated liver volume (ml).

e A_A_ = Activity administered to the patient, at time of administration (MBq).

f A_S_ = Activity at time of SPECT acquisition (MBq).

g A_P_ = Activity at time of PET acquisition (MBq).

### Absorbed Dose Estimation

#### Phantom

The iterative+PSF+TOF PET and iterative+PSF SPECT images of experiments 4 (Table 1) were used to evaluate the accuracy of dosimetry. Calibration factors to convert the PET and SPECT images into units of activity were determined by dividing the total reconstructed counts in the phantom by the known activity in the phantom. A digital phantom representing the ‘true activity distribution’ (TRUE) was constructed from a segmented high-resolution CT dataset (voxel size 0.6×0.6×0.6 mm^3^). Activity concentrations of 1.09 MBq ml^−1^ and 9.6 MBq ml^−1^ were applied to the segmented 3D background ROI and 3D hot sphere ROIs, respectively. Dose maps were calculated from the PET, SPECT, and TRUE activity images by convolution with the appropriate ^90^Y 3D dose-point kernel (DPK), in accordance with MIRD Pamphlet No. 17 [29]. For this purpose, DPKs with the same voxel sizes as the high-resolution CT, SPECT and PET images were calculated using the Monte Carlo engine MCNPX 2.5.0 [30].

Three-dimensional ROIs of the fillable spheres and a 2000 ml background region were delineated on the CT scans, altogether representing a liver with tumours. Cumulative dose-volume histograms (CDVH) and mean absorbed doses were calculated for all ROIs. Additionally, the mean absorbed doses in the hot sphere ROIs were corrected for incomplete activity recovery due to partial volume effects (PVE), by multiplication with the corresponding correction factors calculated as R * C_B_/C_H_. Errors in the uncorrected and corrected SPECT and PET-based dose estimates were calculated in comparison to the TRUE dose.

#### Patients

Patient data were reconstructed with the same settings as the phantom data. Three-dimensional liver ROIs were manually delineated on the CT scans. To be able to mutually compare the SPECT and PET-based dose distributions, the liver ROI of each patient was segmented into a low-dose ROI and a high-dose ROI. The high-dose ROI included all voxels that had on one or both modalities an activity concentration of at least two times the mean liver concentration. The low-dose ROI consisted of the other liver voxels, i.e. the liver ROI excluding the high-dose ROI. The resulting ‘integrated’ SPECT/PET ROIs allowed for an unbiased, direct comparison of the dose distribution between the modalities. Following conversion of PET and SPECT activity images into dose maps, CDVH and mean absorbed doses were calculated for all ROIs.

## Results

### Image Contrast and Noise

**Figure 1:**
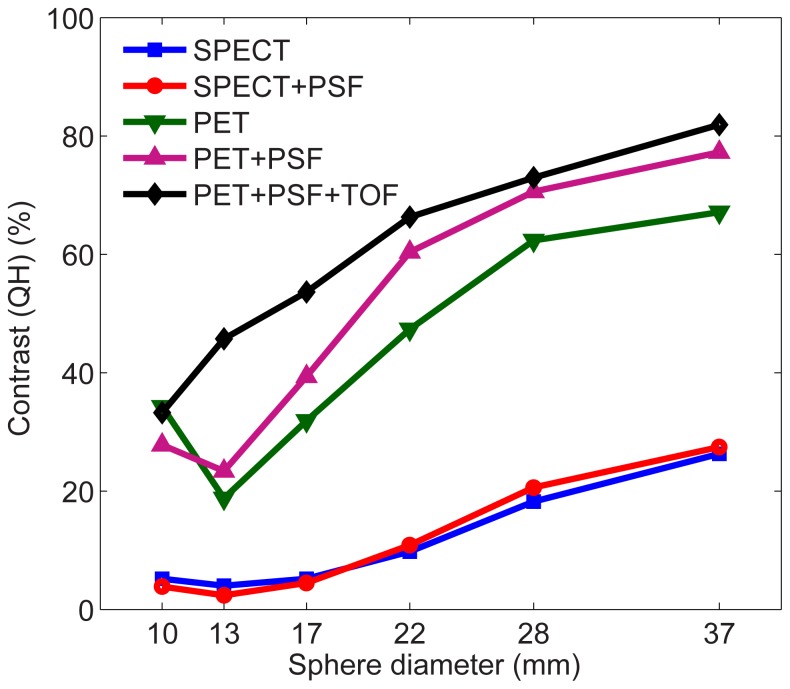
Contrast recovery as a function of sphere diameter for all reconstruction methods.

**Figure 2:**
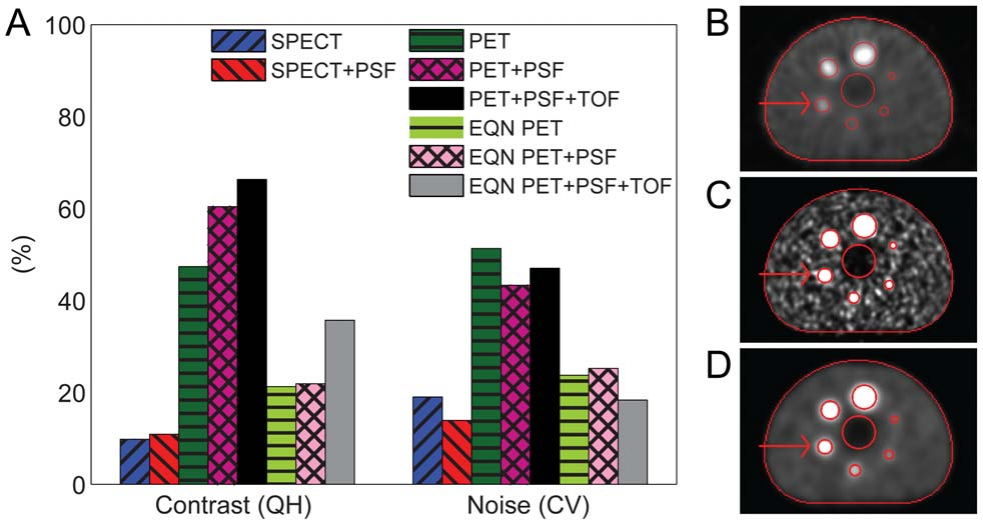
PET and SPECT contrast recovery and image noise. Contrast recovery and image noise in the 22-mm sphere for all reconstruction methods (A), the slice through the center of the SPECT volume (iterative+PSF) (B), the slice through the center of the PET volume (iterative+PSF+TOF) (C), and the slice through the center of the PET volume (iterative+PSF+TOF) reconstructed with SPECT-like noise level (D). All images were linearly window-leveled from 0 to 4 times the mean intensity in the background ROI. The boundaries of the central lung insert, the six hot sphere ROIs, and the background ROI are illustrated by the red lines and the 22-mm sphere is indicated by the arrow.

Figure 1 shows that iterative+PSF+TOF reconstruction resulted in PET images with the highest contrast recovery. Iterative+PSF reconstruction resulted in the highest contrast recovery for SPECT. PET contrast recovery coefficients were substantially higher than those of SPECT in all spheres. In comparison, both image contrast and image noise were higher in PET than in SPECT (Figure 2A), which is also evident from the transversal slices through the phantom in Figure 2B and 2C. The noise level in the PET images was substantially reduced when reconstructed with 1 iteration and a 15 mm FWHM post-reconstruction filter (equal noise (EQN) PET), approximately to the noise level of the SPECT images (approximately 18% CV, Figure 2A and 2D). This set-up allows for a fair comparison of image contrast. At equal noise level, all PET images still showed higher contrast than SPECT, although contrast was lost in comparison with PET images reconstructed with optimal settings.

### Detectability

**Figure 3:**
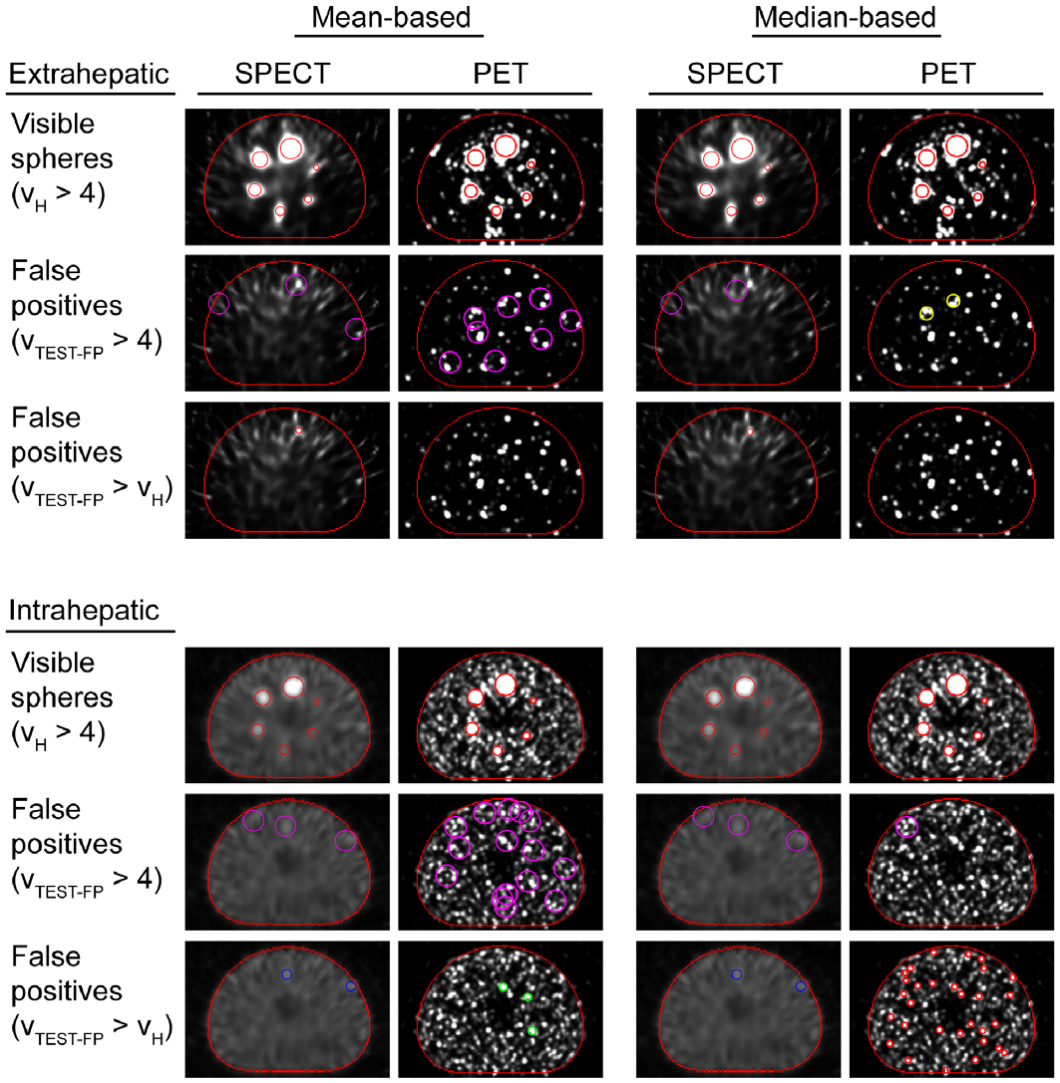
Visualization of the visibility and unique detectability results. The activity concentration in the hot spheres was 2.4 MBq ml^−1^ for both the images without (rows 1–3) and with background activity (rows 4–6). In the 1^st^ and 4^th^ row, the SPECT+PSF (1^st^ and 3^rd^ column) and PET+PSF+TOF (2^nd^ and 4^th^ column) slices are overlaid with the location of the visible phantom spheres (red). In rows 2, 3, 5 and 6 a background slice is overlaid with the location of false positive regions. For illustrative reasons, only the largest false positive ROIs are shown (red: 10 mm; green: 13 mm; blue: 17 mm; yellow: 22 mm; purple: 37 mm). Clustered ROIs with connecting center voxels are represented by the ROI with the highest υ_TEST-FP_ value.

From Table 3 it can be appreciated that the ‘mean-based’ and ‘median-based’ visibility results were equal for all images but one. The unique detectability results differed between the ‘mean-based’ and ‘median-based’ approach, for both detectability criteria. From Figure 3 it is apparent the ‘mean-based’ approach detected more false positives than the ‘median-based’ approach. Being less affected by outliers, the ‘median-based’ approach only detected the more or less uniform false positives regions and was therefore considered the best approach for false positive detection. Consequently, in the remainder of this section the ‘median-based’ results are reported for hot sphere visibility and unique detectability. Table 3 and Figure 3 show that in the extrahepatic situation, all six spheres were visible on SPECT and PET images according to the Rose criterion. According to the criterion υ_TEST-FP_<4, none of the spheres were uniquely detectable with SPECT, whereas the smallest sphere uniquely detectable with PET was 28 mm in diameter. According to the criterion υ_TEST-FP_< υ_H_, all hot spheres in the cold background were uniquely detectable with PET, whereas the smallest sphere uniquely detectable with SPECT was 13 mm in diameter. For all three activity concentrations of the intrahepatic situation, all six spheres were visible with both SPECT and PET, but none of them was uniquely detectable according to the criterion υ_TEST-FP_<4. According to the criterion υ_TEST-FP_< υ_H_, the smallest sphere uniquely detectable with PET was 13 mm in diameter, whereas this was 22 mm for SPECT, for all activity concentrations.

**Table 3:** Extra- and intrahepatic visibility and unique detectibility.

	Extrahepatic	Intrahepatic		
	[A]_sph_ (MBq ml^−1^)	[A]_sph_ (MBq ml^−1^)		
	2.4	9.6	4.8	2.4
**Visible: υ_H_ >4**				
** SPECT**	10 (10)	10 (10)	13 (13)	10 (10)
** SPECT+PSF**	10 (10)	10 (10)	10 (10)	10 (10)
** PET**	10 (10)	10 (10)	10 (10)	10 (13)
** PET+PSF**	10 (10)	10 (10)	10 (10)	10 (10)
** PET+PSF+TOF**	10 (10)	10 (10)	10 (10)	10 (10)
**Uniquely detectable: υ_TEST-FP_<4**				
** SPECT**	X (X)	X (X)	X (X)	X (X)
** SPECT+PSF**	X (X)	X (X)	X (X)	X (X)
** PET**	X (37)	X (X)	X (X)	X (X)
** PET+PSF**	X (X)	X (X)	X (X)	X (X)
** PET+PSF+TOF**	X (28)	X (X)	X (X)	X (X)
**Uniquely detectable: υ_TEST-FP_< υ_H_**				
** SPECT**	17 (17)	22 (22)	22 (22)	22 (28)
** SPECT+PSF**	13 (13)	22 (22)	22 (22)	22 (22)
** PET**	13 (13)	17 (17)	17 (17)	13 (22)
** PET+PSF**	13 (13)	17 (17)	13 (17)	13 (22)
** PET+PSF+TOF**	10 (10)	10 (13)	13 (13)	17 (13)

The extra- and intrahepatic activity distribution is represented by hot spheres in a cold and warm background, respectively. For each image, the diameters (mm) of the first ‘mean-based’ visible and uniquely detectable spheres are given, followed by the diameters of the ‘median-based’ results in brackets. ‘X’ means that none of the spheres were uniquely detectable.

### Absorbed Dose Estimation

#### Phantom

**Figure 4:**
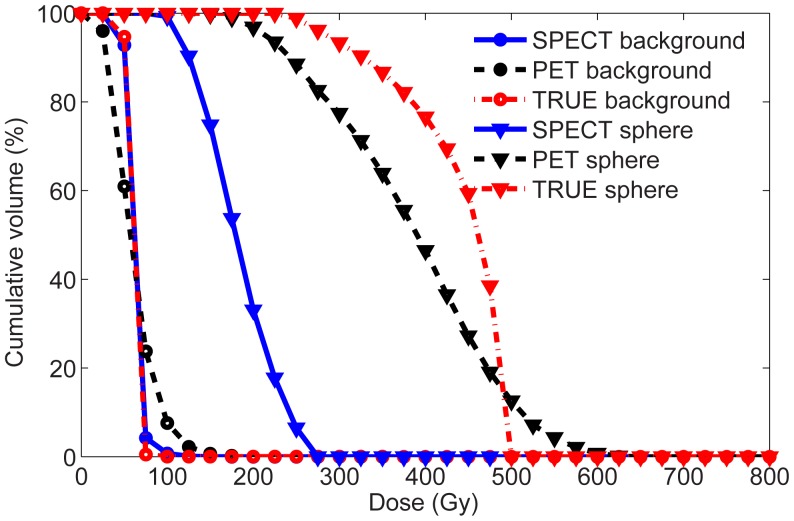
Phantom dosimetry. CDVH of the phantom background ROI and the ROI of the 37-mm diameter sphere. The presented doses were not corrected for PVE.

The error of the uncorrected (in brackets: corrected for PVE) PET dose estimates ranged from −11% (+6%) for the largest sphere to −45% (+40%) for the smallest sphere (Table 4). For SPECT the errors were larger, ranging from −58% (+18%) to −75% (+69%). Figure 4 shows that the PET-based CDVH of the hot sphere ROI followed the true CDVH more closely than the SPECT-based CDVH. The higher image noise in TOF PET was reflected by the flattened slope of the CDVH curve.

**Table 4:** Phantom dosimetry.

	Phantom ROI						
	10	13	17	22	28	37	BKG
**SPECT**	76 (516)	69 (514)	78 (511)	102 (487)	143 (484)	181 (508)	61
**PET**	167 (431)	224 (432)	255 (433)	333 (475)	324 (426)	384 (457)	60
**TRUE**	306	341	372	395	412	432	52

The uncorrected mean dose (Gy) in the 6 hot sphere ROIs and the background ROI (BKG), followed by the mean dose corrected for PVE in brackets (spheres only). The TRUE dose represents the actual dose in the phantom.

#### Patients

**Figure 5:**
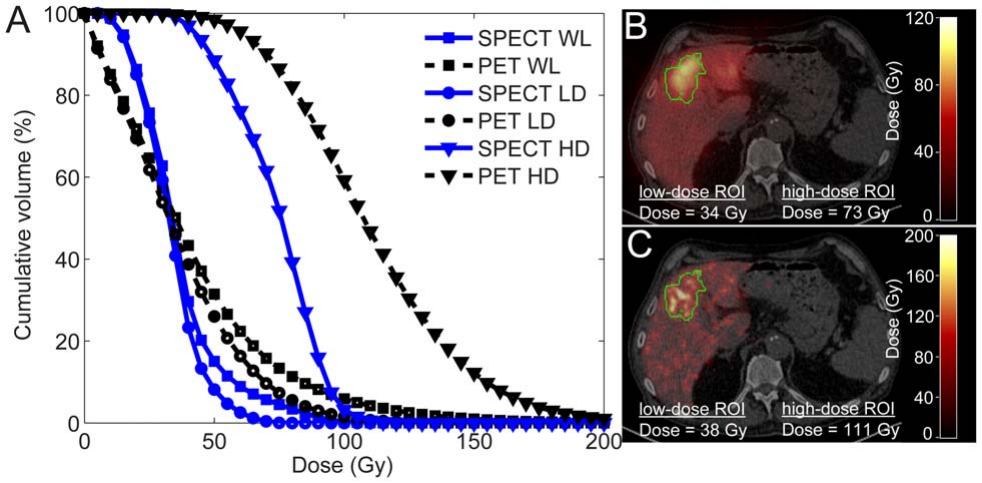
Patient 2 dosimetry. The CDVH of the whole liver (WL), low-dose (LD) and high-dose (HD) ROIs of patient 2 (A), a transversal slice through the SPECT-based dose map, fused with CT (B), and the same transversal slice through the PET-based dose map (C). The boundary of the high-dose ROI is depicted by the green line.

For all patients, the difference between the estimated dose in the high-dose ROI and the low-dose ROI was larger for PET than for SPECT (Table 5). This effect is also illustrated by the CDVH and fused images of patient 2 in Figure 5. Images of patients 1, 3, 4, and 5 are provided as Figure S1, Figure S2, Figure S3 and Figure S4, respectively.

**Table 5:** Patient dosimetry.

	Patient ROI									
	1		2		3		4		5	
	LD	HD	LD	HD	LD	HD	LD	HD	LD	HD
**SPECT**	26	37	34	73	30	53	22	36	12	31
**PET**	29	85	38	111	33	80	26	58	11	47

The mean dose (Gy) in the low-dose ROI (LD) and the high-dose ROI (HD) are given for all patients.

## Discussion

In this study, we quantitatively compared the accuracy of ^90^Y Bremsstrahlung SPECT and state-of-the-art PET for the assessment of the intra- and extrahepatic microsphere distribution after radioembolization, using the physical parameters image contrast and image noise, and the clinically relevant parameters detectability and dosimetry. We realize that image contrast and noise not only depend on the fundamental limitations of the imaging modalities, but also on acquisition and reconstruction settings; e.g., a well-known property of iterative reconstruction algorithms is that contrast can be maximized at the cost of noise amplification by varying the number of iterations [31]. Before defining the study setup as published, we evaluated image contrast and noise for various energy windows (wide: 50–250 keV [6], [9], [23], [32]–[35]; medium-wide: 105–195 keV [8], [24]; small: 76–105 keV [36], [37], SPECT only), various numbers of iterations (1, 3, 5, and 8), and various post-reconstruction filters (0, 5, and 10 mm FWHM). The settings resulting in the highest image contrast were the starting point for this study. The results imply that ^90^Y Bremsstrahlung SPECT and PET image contrast is improved by inclusion of a PSF model in the reconstruction process. The PET image contrast is further improved by inclusion of TOF information, which also resulted in slightly higher image noise. The latter can be attributed to faster convergence of TOF reconstruction [20]. At similar noise level, all PET algorithms demonstrated higher contrast recovery coefficients than optimal SPECT. Therefore, we conclude that the image quality of ^90^Y state-of-the-art PET outperforms Bremsstrahlung SPECT.

In comparison with ^18^F PET and ^99m^Tc SPECT, the Q_H_ of ^90^Y were consistently lower (e.g. 66% (^90^Y) vs 71% (^18^F) and 11% (^90^Y) vs 57% (^99m^Tc) for the 22-mm sphere, data not shown). The large difference in SPECT Q_H_ can probably be attributed to the low spatial resolution and the large amount of photon scatter and septal penetration with ^90^Y [9], all of which increase the PVE. Recent work on ^90^Y SPECT reconstruction demonstrated that image contrast may be substantially improved by including accurate models of the scattered and penetrated photons, but these algorithms are not available in the clinic to date [8], [10]. To the contrary, PET reconstruction algorithms include advanced techniques to correct for random coincidences, photon scatter, and attenuation effects. Nevertheless, a slight difference in Q_H_ between ^90^Y and ^18^F was observed, which was probably not caused by resolution effects; the difference in spatial resolution was estimated to be within 1%, using the analytical resolution model of Lubberink and Herzog [38]. Instead, the intensity in the low-count background region may have been overestimated, possibly due to sparsity of the (smoothed) delayed coincidences sinogram that is used for random correction in the OP-OSEM algorithm [39]. This effect has also been observed in dynamic studies with short frame rates [40]. Further research is required to fully understand the clinical impact of these effects.

Inadvertent extrahepatic deposition of activity may be detected directly post-treatment on ^90^Y SPECT or PET images. Ahmadzadehfar *et al* showed that detection of extrahepatic activity on Bremsstrahlung SPECT/CT scans predicted GI ulcers with a sensitivity of 87% and a specificity of 100%, despite low image quality [6]. In this study, we investigated the detectability of accumulations of activity of different sizes with the well-known Rose criterion for detection of false negatives and with a new method for detection of false positives. The ‘median-based’ unique detectability approach was deemed better than its ‘mean-based’ equivalent, since the latter tended to erroneously classify non-uniform regions as ‘false positive’. We demonstrated that the visibility of intra- and extrahepatic accumulations of activity was equal between SPECT and PET, for the investigated sphere sizes and activity concentration ratios. Unique detectability with the υ_TEST-FP_< υ_H_ criterion demonstrated larger differences between PET and SPECT than the υ_TEST-FP_<4 criterion, but will depend on the activity concentration in the hot spheres. Nevertheless, according to both criteria, PET was able to uniquely detect smaller accumulations of activity than SPECT, endorsing the use of (TOF) PET/CT instead of SPECT/CT. Given the relative simplicity of the detectability models used, we should emphasize that the results of our phantom study should not be interpreted as the lower limits of (lesion) detection performance by a human observer, but merely as a comparison between ^90^Y SPECT and PET. The results are likely to be dependent on acquisition and reconstruction settings, which were optimized for image contrast in this study. Optimization of the imaging protocols for noise, e.g. by increasing the FWHM of the post-reconstruction filter, may improve the unique detectability of hot spots.

Accurate tumour and liver dosimetry can improve RE patient care, e.g. by facilitating (selective) re-treatment of tumourous tissue that did not receive a sufficient dose during the first treatment. The relationship between the ^99m^Tc-MAA SPECT-based tumour absorbed dose and tumour response is reported in numerous publications investigating the possibilities of treatment optimization by individualized ^99m^Tc-MAA SPECT-based dose planning (e.g. [7], [33], [41]). To the contrary, publications on the ^90^Y SPECT or PET-based dose-response relation are relatively sparse [42], [43]. The latter approach has the advantage of imaging the real ^90^Y microsphere distribution, which avoids the question of accurate prediction of the microsphere distribution with ^99m^Tc-MAA, but it is not used for individualized treatment planning. Using phantom experiments, we demonstrated that ^90^Y TOF PET-based dose estimates were more accurate than Bremsstrahlung SPECT-based dose estimates. Based on the incomplete contrast recovery observed in the image quality phantom, dose underestimations with both PET and SPECT were to be expected. SPECT-based absorbed dose estimates in the high-dose regions of the phantom were lower than those of PET, which was supported by the results of our patient study. When correcting the hot sphere mean dose estimates for PVE, the absolute dose errors were reduced, but dose overestimations up to 69% were still observed. The use of PVE correction factors is restricted to correction of mean ROI values, and PVE correction was therefore applied to the mean absorbed dose values of the hot sphere ROIs. Due to the range of the beta-particles, however, the mean absorbed dose in the hot sphere ROIs not only depends on the locally absorbed energy as a result of activity in the ROIs itself, but also on the energy absorbed as a result of activity in adjacent background voxels. With hot spheres in a warm background, PVE result in an underestimation of the first contribution to the mean absorbed dose, whereas the latter contribution is overestimated. Consequently, application of the activity-based PVE correction factors leads to erroneous up-scaling of the adjacent background voxels’ contribution to the mean absorbed dose in the hot sphere ROIs. This effect is stronger for smaller spheres, due to the relatively large amount of adjacent background voxels, and stronger for SPECT than for PET, due to the higher correction factors of the first. For dosimetry purposes, a better approach may be to determine correction factors on dose images, which not only include PVE but also DPK effects. However, these dose correction factors will strongly depend on the activity concentration ratio between ROIs and adjacent background voxels, which undesirably complicates PVE correction in clinical practice. For this reason, we chose not to correct the mean dose estimates in the patient study for PVE effects. The results of the patient study indicate that the use of high-resolution PET can potentially improve dose-response relationships on the level of the tumour for ^90^Y RE. The evaluation of the estimated absorbed liver dose in low-dose and high-dose regions, which did not necessarily coincide with healthy liver and tumour regions, did not harm the conclusions presented in this study, since the primary aim was a quantitative comparison of the ^90^Y SPECT and PET-based dose distribution, and not clinical liver dosimetry. Evaluation of the absorbed dose to tumourous and healthy liver tissue should be performed in a larger (prospective) patient study and is beyond the scope of this study.

### Conclusion

This study demonstrates that the ^90^Y image quality of state-of-the-art PET is superior over Bremsstrahlung SPECT for the assessment of the microsphere distribution after radioembolization. Intra- and extrahepatic hot spots of 10 mm in diameter or larger are visible on SPECT and PET images, but PET is able to uniquely detect smaller accumulations of activity than SPECT. Additionally, TOF PET-based dose estimates are more accurate than SPECT-based dose estimates, which give large underestimations in high-dose regions.

## Supporting Information

Figure S1 **Patient 1 dosimetry.** The CDVH of the whole liver (WL), low-dose (LD) and high-dose (HD) ROIs of patient 1 (A), a transversal slice through the SPECT-based dose map, fused with CT (B), and the same transversal slice through the PET-based dose map (C). The boundary of the high-dose ROI is depicted by the green line.
(TIF)

Figure S2 **Patient 3 dosimetry.** The CDVH of the whole liver (WL), low-dose (LD) and high-dose (HD) ROIs of patient 3 (A), a transversal slice through the SPECT-based dose map, fused with CT (B), and the same transversal slice through the PET-based dose map (C). The boundary of the high-dose ROI is depicted by the green line.
(TIF)

Figure S3 **Patient 4 dosimetry.** The CDVH of the whole liver (WL), low-dose (LD) and high-dose (HD) ROIs of patient 4 (A), a transversal slice through the SPECT-based dose map, fused with CT (B), and the same transversal slice through the PET-based dose map (C). The boundary of the high-dose ROI is depicted by the green line.
(TIF)

Figure S4 **Patient 5 dosimetry.** The CDVH of the whole liver (WL), low-dose (LD) and high-dose (HD) ROIs of patient 5 (A), a transversal slice through the SPECT-based dose map, fused with CT (B), and the same transversal slice through the PET-based dose map (C). The boundary of the high-dose ROI is depicted by the green line.
(TIF)

## References

[1] Vente MA, Wondergem M, van der Tweel I, van den Bosch MA, Zonnenberg BA, et al (2009) Yttrium-90 microsphere radioembolization for the treatment of liver malignancies: a structured meta-analysis. Eur Radiol 19: 951–959. 18989675 10.1007/s00330-008-1211-7

[2] Kennedy AS, Salem R (2010) Radioembolization (yttrium-90 microspheres) for primary and metastatic hepatic malignancies. Cancer J 16: 163–175. 20404614 10.1097/PPO.0b013e3181d7e8cf

[3] Kennedy AS, Nag S, Salem R, Murthy R, Mcewan AJ, et al (2007) Recommendations for radioembolization of hepatic malignancies using yttrium-90 microsphere brachytherapy: A consensus panel report from the Radioembolization Brachytherapy Oncology Consortium. Int J Radiat Oncol Biol Phys 68: 13–23. 17448867 10.1016/j.ijrobp.2006.11.060

[4] Murthy R, Brown DB, Salem R, Meranze SG, Coldwell DM, et al (2007) Gastrointestinal complications associated with hepatic arterial yttrium-90 microsphere therapy. J Vasc Interv Radiol 18: 553–562. 17446547 10.1016/j.jvir.2007.02.002

[5] Riaz A, Lewandowski RJ, Kulik LM, Mulcahy MF, Sato KT, et al (2009) Complications Following Radioembolization with Yttrium-90 Microspheres: A Comprehensive Literature Review. J Vasc Interv Radiol 20: 1121–1130. 19640737 10.1016/j.jvir.2009.05.030

[6] Ahmadzadehfar H, Muckle M, Sabet A, Kuhl C, Biermann K, et al (2010) The significance of Bremsstrahlung SPECT-CT of the Abdomen after Yttrium 90 microsphere selective internal radiation treatment (SIRT) in the early Diagnosis of SIRT-Induced Extrahepatic Side Effects. Eur J Nucl Med Mol Imaging 37: S264–S264.

[7] Chiesa C, Maccauro M, Romito R, Spreafico C, Pellizzari S, et al (2011) Need, feasibility and convenience of dosimetric treatment planning in liver selective internal radiation therapy with Y-90 microspheres: the experience of the National Cancer Institute of Milan. Q J Nucl Med Mol Imag 55: 168–197. 21386789

[8] Minarik D, Sjogreen GK, Ljungberg M (2008) Evaluation of quantitative (90)Y SPECT based on experimental phantom studies. Phys Med Biol 53: 5689–5703. 18812648 10.1088/0031-9155/53/20/008

[9] Elschot M, Nijsen JFW, Dam AJ, de Jong HWAM (2011) Quantitative Evaluation of Scintillation Camera Imaging Characteristics of Isotopes Used in Liver Radioembolization. PLoS ONE 6: e26174. 22073149 10.1371/journal.pone.0026174 PMC3207835

[10] Rong X, Du Y, Ljungberg M, Rault E, Vandenberghe S, et al (2012) Development and evaluation of an improved quantitative Y-90 bremsstrahlung SPECT method. Med Phys 39: 2346–2358. 22559605 10.1118/1.3700174 PMC3338590

[11] Lhommel R, van Elmbt L, Goffette P, Van den Eynde M, Jamar F, et al (2010) Feasibility of (90)Y TOF PET-based dosimetry in liver metastasis therapy using SIR-Spheres. Eur J Nucl Med Mol Imaging 37: 1654–1662. 20422185 10.1007/s00259-010-1470-9

[12] Langhoff H, Hennies HH (1961) Zum Experimentellen Nachweis Von Zweiquantenzerfall Beim 0+-0+-Ubergang des Zr90. Z Phys A At Nucl 164: 166–173.

[13] Nickles RJ, Roberts AD, Nye JA, Converse AK, Barnhart TE, et al.. (2004) Assaying and PET imaging of yttrium-90: 1>34ppm>0. Conf Record of IEEE Nuclear Science Symp and Medical Imaging Conf. 3412–3414.

[14] Bailey DL, Karp JS, Surti S (2003) Physics and instrumentation in PET. In: Valk PE, Bailey DL, Townsend DW, Maisey MN. Positron Emission Tomography: Basic Science and Clinical Practice. London: Springer-Verlag. 41–67.

[15] van Elmbt L, Walrand S, Lhommel R, Jamar F, Pauwels S (2010) Quantitative comparison between LYSO and BGO PET-tomographs in 90Y imaging. Eur J Nucl Med Mol Imaging 37: S293–S293. 10.1007/s00259-010-1470-9 20422185

[16] Lhommel R, Goffette P, Van den Eynde M, Jamar F, Pauwels S, et al (2009) Yttrium-90 TOF PET scan demonstrates high-resolution biodistribution after liver SIRT. Eur J Nucl Med Mol Imaging 36: 1696–1696. 19618182 10.1007/s00259-009-1210-1

[17] Werner MK, Brechtel K, Beyer T, Dittmann H, Pfannenberg C, et al (2010) PET/CT for the assessment and quantification of Y-90 biodistribution after selective internal radiotherapy (SIRT) of liver metastases. Eur J Nucl Med Mol Imaging 37: 407–408. 19997914 10.1007/s00259-009-1317-4

[18] Gates VL, Esmail AAH, Marshall K, Spies S, Salem R (2011) Internal Pair Production of (90)Y Permits Hepatic Localization of Microspheres Using Routine PET: Proof of Concept. J Nucl Med 52: 72–76. 21149493 10.2967/jnumed.110.080986

[19] D’Arienzo M, Chiaramida P, Chiacchiararelli L, Coniglio A, Cianni R, et al (2012) Y-90 PET-based dosimetry after selective internal radiotherapy treatments. Nuc Med Comm 33: 633–640. 10.1097/MNM.0b013e3283524220 22407156

[20] Jakoby BW, Bercier Y, Conti M, Casey ME, Bendriem B, et al (2011) Physical and clinical performance of the mCT time-of-flight PET/CT scanner. Phys Med Biol 56: 2375–2389. 21427485 10.1088/0031-9155/56/8/004

[21] Comtat C, Kinahan PE, Defrise M, Michel C, Townsend DW (1998) Fast reconstruction of 3D PET data with accurate statistical modeling. IEEE Trans Nucl Sci 45: 1083–1089.

[22] Panin VY, Kehren F, Michel C, Casey M (2006) Fully 3-D PET reconstruction with system matrix derived from point source measurements. IEEE Trans Med Imag 25: 907–921. 10.1109/tmi.2006.876171 16827491

[23] Shen S, DeNardo GL, Yuan A, DeNardo DA, DeNardo SJ (1994) Planar gamma camera imaging and quantitation of yttrium-90 bremsstrahlung. J Nucl Med 35: 1381–1389. 8046498

[24] Minarik D, Ljungberg M, Segars P, Gleisner KS (2009) Evaluation of quantitative planar 90Y bremsstrahlung whole-body imaging. Phys Med Biol 54: 5873–5883. 19759410 10.1088/0031-9155/54/19/014

[25] Tsui BMW, Hu HB, Gilland DR, Gullberg GT (1988) Implementation of Simultaneous Attenuation and Detector Response Correction in Spect. IEEE Trans Nucl Sci 35: 778–783.

[26] National Electrical Manufacturers Association (2007) NEMA Standards Publication NU 2–2007: Performance Measurements of Positron Emission Tomographs. Rosslyn, VA: National Electrical Manufacturers Association.

[27] Cherry SR., Sorenson JA, Phelps ME (2003) Physics in Nuclear Medicine. Philadelphia: Saunders. 290.

[28] Rose A (1948) The Sensitivity Performance of the Human Eye on An Absolute Scale. J Opt Soc Am 38: 196–208. 18901781 10.1364/josa.38.000196

[29] Bolch WE, Bouchet LG, Robertson JS, Wessels BW, Siegel JA, et al (1999) MIRD pamphlet No. 17: the dosimetry of nonuniform activity distributions–radionuclide S values at the voxel level. Medical Internal Radiation Dose Committee. J Nucl Med 40: 11S–36S. 9935083

[30] Hendricks JS, McKinney GW, Waters LS, Roberts TS, Egdorf HW et al. (2005) MCNPX Extensions, Version 2.5.0. Los Alamos, CA: Los Alamos National Laboratory Report LA-UR-05-2675.

[31] Hutton BF, Hudson HM, Beekman FJ (1997) A clinical perspective of accelerated statistical reconstruction. Eur J Nucl Med Mol Imaging 24: 797–808. 10.1007/BF00879671 9211768

[32] Clarke LP, Cullom SJ, Shaw R, Reece C, Penney BC, et al (1992) Bremsstrahlung Imaging Using the Gamma-Camera - Factors Affecting Attenuation. J Nucl Med 33: 161–166. 1730984

[33] Flamen P, Vanderlinden B, Delatte P, Ghanem G, Ameye L, et al (2008) Multimodality imaging can predict the metabolic response of unresectable colorectal liver metastases to radioembolization therapy with Yttrium-90 labeled resin microspheres. Phys Med Biol 53: 6591–6603. 18978442 10.1088/0031-9155/53/22/019

[34] Fabbri C, Sarti G, Cremonesi M, Ferrari M, Di DA, et al (2009) Quantitative analysis of 90Y Bremsstrahlung SPECT-CT images for application to 3D patient-specific dosimetry. Cancer Biother Radiopharm 24: 145–154. 19243257 10.1089/cbr.2008.0543

[35] Ito S, Kurosawa H, Kasahara H, Teraoka S, Ariga E, et al (2009) (90)Y bremsstrahlung emission computed tomography using gamma cameras. Ann Nucl Med 23: 257–267. 19326187 10.1007/s12149-009-0233-9

[36] Mansberg R, Sorensen N, Mansberg V, Van der Wall H (2007) Yttrium 90 Bremsstrahlung SPECT/CT scan demonstrating areas of tracer/tumour uptake. Eur J Nucl Med Mol Imaging 34: 1887. 17846767 10.1007/s00259-007-0536-9

[37] Knesaurek K, Machac J, Muzinic M, DaCosta M, Zhang Z, et al (2010) Quantitative comparison of yttrium-90 (90Y)-microspheres and technetium-99m (99mTc)-macroaggregated albumin SPECT images for planning 90Y therapy of liver cancer. Technol Cancer Res Treat 9: 253–262. 20441235 10.1177/153303461000900304

[38] Lubberink M, Herzog H (2011) Quantitative imaging of (124)I and (86)Y with PET. Eur J Nucl Med Mol Imaging 38: 10–18. 10.1007/s00259-011-1768-2 PMC3098993 21484385

[39] Comtat C, Bataille F, Michel C, Jones JP, Sibomana M, et al.. (2004) OSEM-3D reconstruction strategies for the ECAT HRRT. Conf Record of IEEE Nuclear Science Symp and Medical Imaging Conf. 3492–3496.

[40] van Velden FHP, Kloet RW, van Berckel BNM, Wolfensberger SPA, Lammertsma AA, et al (2008) Comparison of 3D-OP-OSEM and 3D-FBP reconstruction algorithms for High-Resolution Research Tomograph studies: effects of randoms estimation methods. Phys Med Biol 53: 3217–3230. 18506070 10.1088/0031-9155/53/12/010

[41] Garin E, Lenoir L, Rolland Y, Edeline J, Mesbah H, et al (2012) Dosimetry Based on Tc-99m-Macroaggregated Albumin SPECT/CT Accurately Predicts Tumor Response and Survival in Hepatocellular Carcinoma Patients Treated with Y-90-Loaded Glass Microspheres: Preliminary Results. J Nucl Med 53: 255–263. 22302962 10.2967/jnumed.111.094235

[42] Strigari L, Sciuto R, Rea S, Carpanese L, Pizzi G, et al (2010) Efficacy and Toxicity Related to Treatment of Hepatocellular Carcinoma with Y-90-SIR Spheres: Radiobiologic Considerations. J Nucl Med 51: 1377–1385. 20720056 10.2967/jnumed.110.075861

[43] Walrand S, Lhommel R, Goffette P, Van den Eynde M, Pauwels S, et al (2012) Hemoglobin level significantly impacts the tumor cell survival fraction in humans after internal radiotherapy. EJNMMI Research 2: 20. 22608186 10.1186/2191-219X-2-20 PMC3413597

