# Supplementary figures and images for: Quantitative Comparison of PET and Bremsstrahlung SPECT for Imaging the In Vivo Yttrium-90 Microsphere Distribution after Liver Radioembolization
Source: PLoS One. 2013 Feb 6;8(2):e55742. doi: 10.1371/journal.pone.0055742 (PMC3566032; doi:10.1371/journal.pone.0055742)

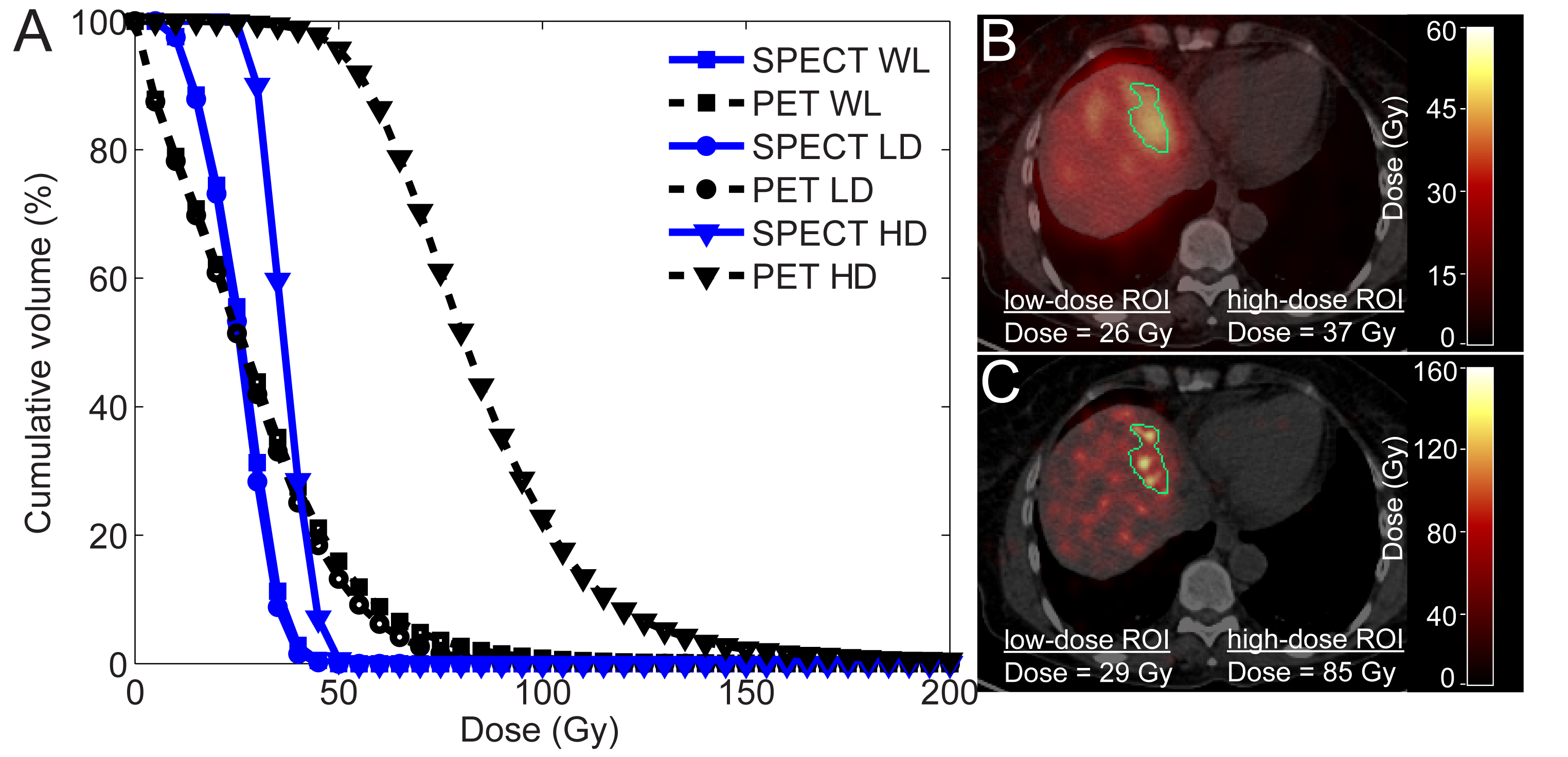

Supplement: Figure S1 — Patient 1 dosimetry. The CDVH of the whole liver (WL), low-dose (LD) and high-dose (HD) ROIs of patient 1 (A), a transversal slice through the SPECT-based dose map, fused with CT (B), and the same transversal slice through the PET-based dose map (C). The boundary of the high-dose ROI is depicted by the green line. (TIF) [file pone.0055742.s001.tif]

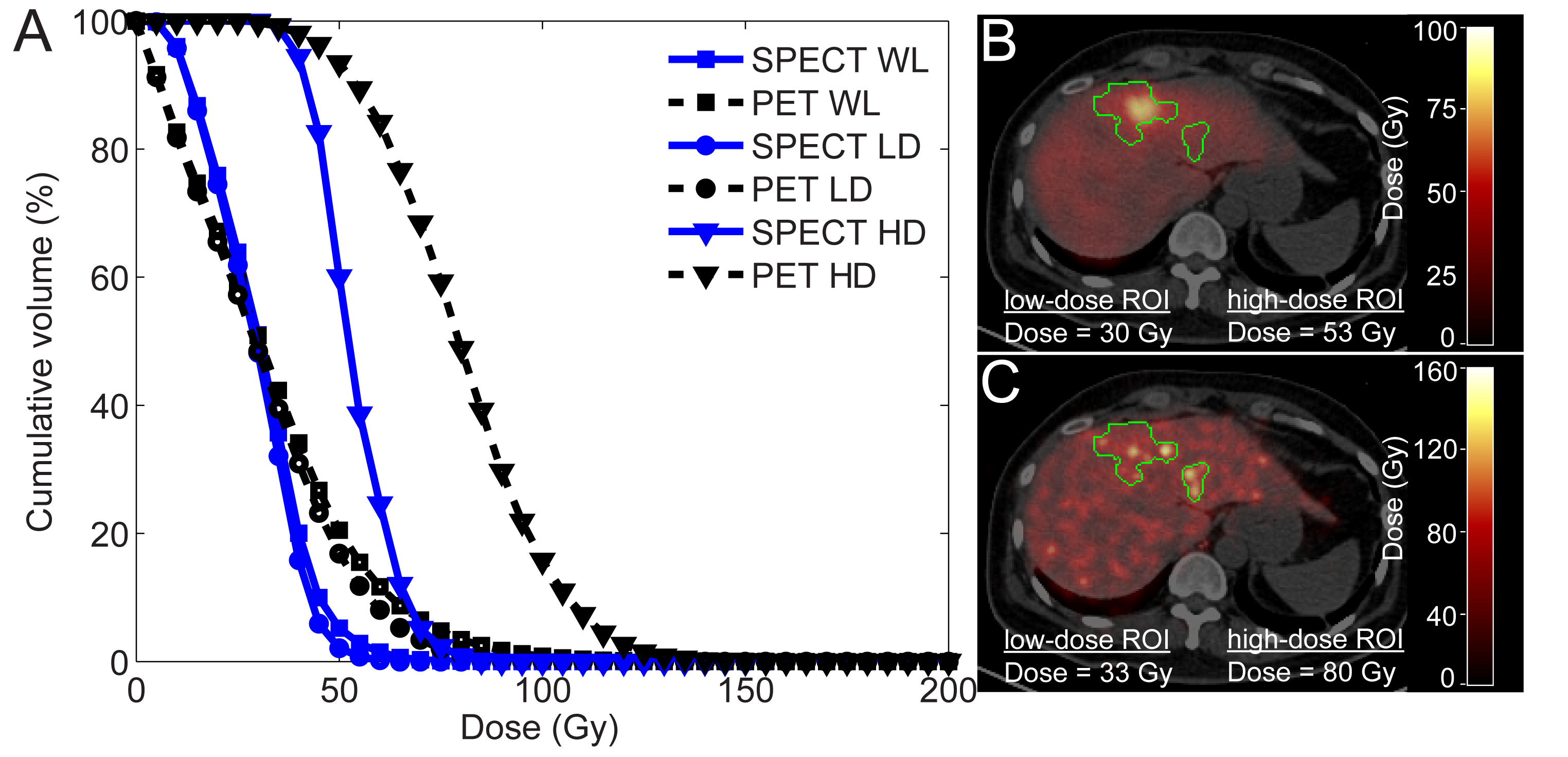

Supplement: Figure S2 — Patient 3 dosimetry. The CDVH of the whole liver (WL), low-dose (LD) and high-dose (HD) ROIs of patient 3 (A), a transversal slice through the SPECT-based dose map, fused with CT (B), and the same transversal slice through the PET-based dose map (C). The boundary of the high-dose ROI is depicted by the green line. (TIF) [file pone.0055742.s002.tif]

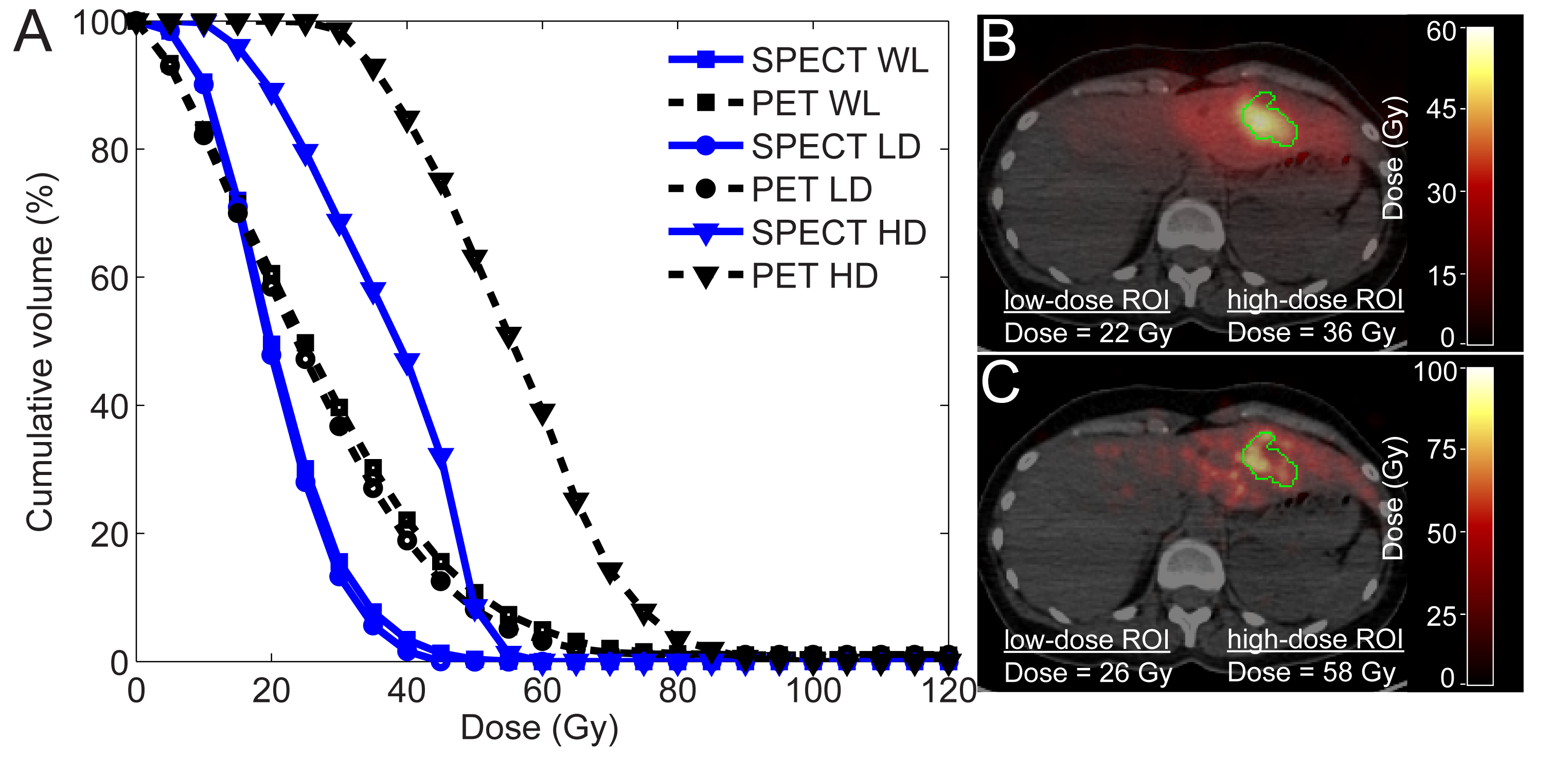

Supplement: Figure S3 — Patient 4 dosimetry. The CDVH of the whole liver (WL), low-dose (LD) and high-dose (HD) ROIs of patient 4 (A), a transversal slice through the SPECT-based dose map, fused with CT (B), and the same transversal slice through the PET-based dose map (C). The boundary of the high-dose ROI is depicted by the green line. (TIF) [file pone.0055742.s003.tif]

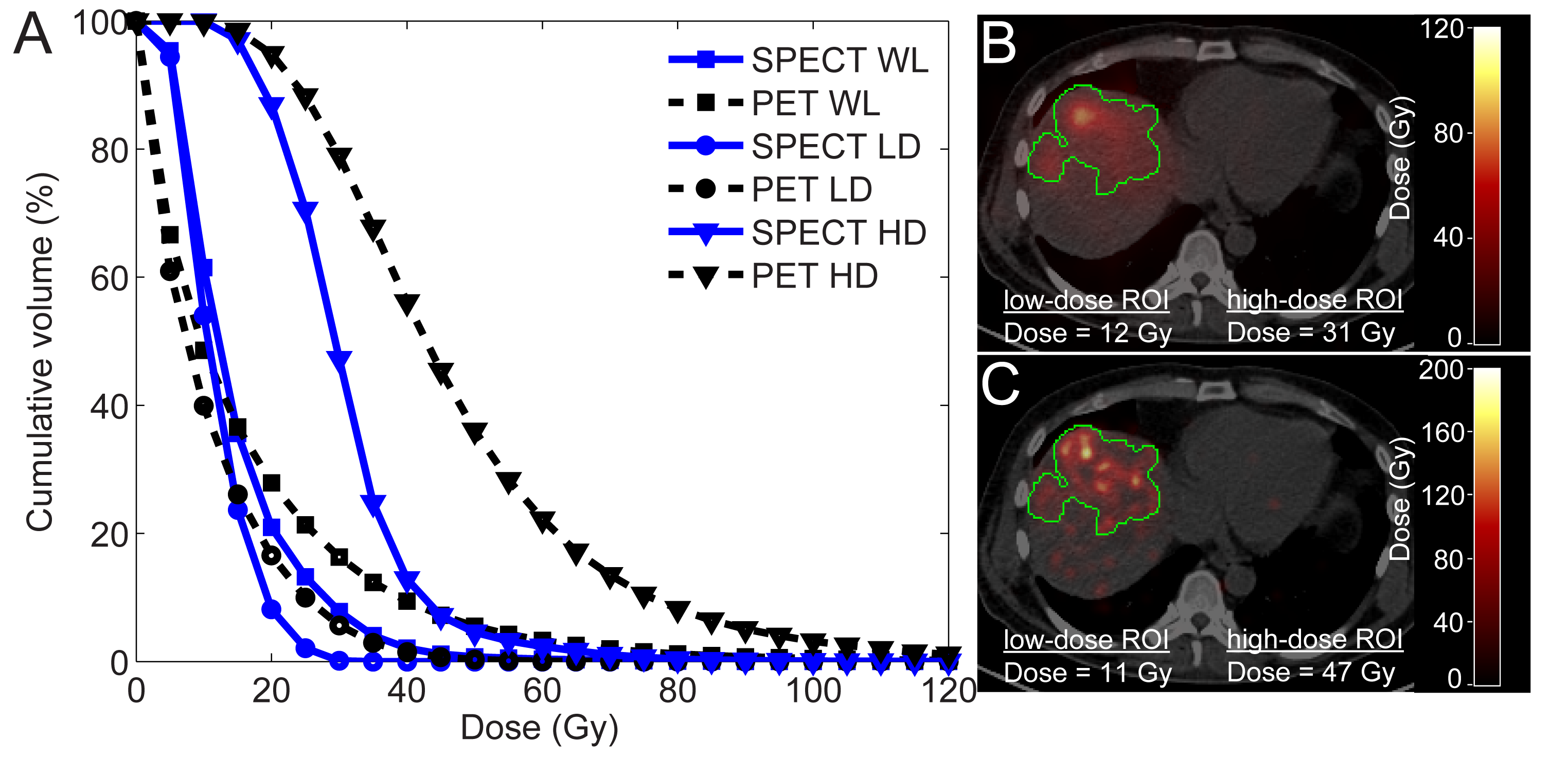

Supplement: Figure S4 — Patient 5 dosimetry. The CDVH of the whole liver (WL), low-dose (LD) and high-dose (HD) ROIs of patient 5 (A), a transversal slice through the SPECT-based dose map, fused with CT (B), and the same transversal slice through the PET-based dose map (C). The boundary of the high-dose ROI is depicted by the green line. (TIF) [file pone.0055742.s004.tif]
